# Androgen Signaling Promotes Translation of *TMEFF2* in Prostate Cancer Cells via Phosphorylation of the α Subunit of the Translation Initiation Factor 2

**DOI:** 10.1371/journal.pone.0055257

**Published:** 2013-02-06

**Authors:** Ryan F. Overcash, Vesna A. Chappell, Thomas Green, Christopher B. Geyer, Adam S. Asch, Maria J. Ruiz-Echevarría

**Affiliations:** 1 Department of Biochemistry and Molecular Biology, Brody School of Medicine at East Carolina University, Greenville, North Carolina, United States of America; 2 Department of Anatomy and Cell Biology, Brody School of Medicine at East Carolina University, Greenville, North Carolina, United States of America; 3 Department of Internal Medicine, Division of Hematology/Oncology. Brody School of Medicine at East Carolina University, Greenville, North Carolina, United States of America; 4 Lineberger Comprehensive Cancer Center, University of North Carolina at Chapel Hill, Chapel Hill, North Carolina, United States of America; University of Colorado, United States of America

## Abstract

The type I transmembrane protein with epidermal growth factor and two follistatin motifs 2 (TMEFF2), is expressed mainly in brain and prostate. Expression of TMEFF2 is deregulated in prostate cancer, suggesting a role in this disease, but the molecular mechanism(s) involved in this effect are not clear. Although androgens promote *tmeff2* transcription, androgen delivery to castrated animals carrying CWR22 xenografts increases TMEFF2 protein levels in the absence of mRNA changes, suggesting that *TMEFF2* may also be post-transcriptionally regulated. Here we show that translation of *TMEFF2* is regulated by androgens. Addition of physiological concentrations of dihydrotestosterone (DHT) to prostate cancer cell lines increases translation of endogenous *TMEFF2* or transfected *TMEFF2-Luciferase* fusions, and this effect requires the presence of upstream open reading frames (uORFs) in the 5′-untranslated region (5′-UTR) of *TMEFF2*. Using chemical and siRNA inhibition of the androgen receptor (AR), we show that the androgen effect on *TMEFF2* translation is mediated by the AR. Importantly, DHT also promotes phosphorylation of the α subunit of the translation initiation factor 2 (eIF2α) in an AR-dependent manner, paralleling the effect on *TMEFF2* translation. Moreover, endoplasmic reticulum (ER) stress conditions, which promote eIF2α phosphorylation, also stimulate *TMEFF2* translation. These results indicate that androgen signaling promotes eIF2α phosphorylation and subsequent translation of *TMEFF2* via a mechanism that requires uORFs in the 5′-UTR of *TMEFF2*.

## Introduction

Androgens signaling through the AR play an essential role in normal prostate development and contribute to the progression of prostate cancer. Binding of androgens to the AR promotes a conformational change that ultimately leads to its translocation to the nucleus and regulation of transcription of a specific set of androgen-responsive genes. Clinical and experimental evidence suggest that prostate cancer progression occurs through alteration of the normal androgen signaling, reducing the specificity or the amount of AR ligand required for proliferation and survival [1]. Importantly, recent results indicate that the function of the AR is specific to the disease stage, triggering a different gene expression program in androgen-dependent as compared to androgen-independent prostate cancer [2]. While the role of the AR signaling axis in transcriptional regulation is well documented, very little is known regarding its role in translation initiation proposed in early studies [3], [4].

The transmembrane protein with epidermal growth factor and two follistatin motifs 2 (TMEFF2) is a single pass transmembrane protein. TMEFF2 is expressed in the embryo [5], [6] and selectively in the adult brain and prostate [7]–[9]. A role for TMEFF2 in prostate cancer was suggested by studies indicating that TMEFF2 expression is altered in a significant fraction of primary and metastatic prostate tumors [5]–[10]. In addition, we recently demonstrated that TMEFF2 interacts with sarcosine dehydrogenase (SARDH), the enzyme responsible for conversion of sarcosine to glycine [11]. Importantly, sarcosine was identified as a marker for prostate cancer progression in a large-scale screen of metabolites from human prostate samples [12]. Increased plasma and urine sarcosine levels distinguished prostate cancer from benign prostate tissue, and were further elevated in metastatic cancer. In addition, sarcosine metabolism and the enzymes involved in it (i.e. SARDH) were shown to act as regulators of cell invasion and metastasis [12]. Therefore, the interaction of TMEFF2 with SARDH further suggests a role for TMEFF2 in prostate cancer progression. In fact, we have also established that full-length TMEFF2 functions as a tumor suppressor and that this role correlates, at least in part, with its ability to interact with SARDH and modulate the cellular levels of sarcosine [11].

In this study we report that translation of *TMEFF2* is regulated by androgens, and this effect requires a functional AR. Results using xenograft models and prostate cancer cell lines established that TMEFF2 expression changes in response to androgens and/or the androgen-dependent or -independent condition of the cells [8], [10]. As demonstrated by Gery et al., [8] these changes are in part due to transcriptional activation of *tmeff2* in response to androgens. However, increased TMEFF2 protein levels in the absence of a corresponding increase in mRNA levels have been observed after addition of androgens to castrated animals carrying CWR22 xenografts, suggesting that *TMEFF2* may also be post-transcriptionally regulated [10].

The *TMEFF2* mRNA has several potential upstream open reading frames (uORFs) in its leader region, and sequence analysis suggests that they are well conserved among mammals. Although only present in 5–10% of the cellular mRNAs, uORFs are common in the leader regions of mRNAs encoding oncoproteins or proteins involved in the control of cellular growth and differentiation, and they function by modulating translation of these essential genes [13]. After being translated, uORFs generally block translation of the main downstream coding region by hampering translation reinitiation at the main translation initiation codon. However, uORFs can promote selective translation of the downstream coding region under cellular stress or other conditions that increase phosphorylation of the α subunit of the eukaryotic translation initiation factor 2 (eIF2α) [13].

eIF2 in its GTP-bound form is required for the selection of the translation initiation codon. Phosphorylation of the α subunit of eIF2 at Ser-51 (eIF2α-P) inhibits the exchange of eIF2-GDP to eIF2-GTP, preventing recognition of the initiating codon and decreasing global translation initiation [14]. However, as mentioned above, uORF-containing mRNAs are actively translated under these conditions. Two mechanisms have been proposed to explain this effect. In the first one, exemplified by the *ATF4* mRNA that contains two uORFs, translation reinitiation at the inhibitory downstream uORF is bypassed under conditions of eIF2α-P, due to the fact that the lower levels of eIF2-GTP increase the time required for the scanning ribosomes to re-acquire eIF2-GTP and reinitiate translation [15]. In the second one, observed in mRNAs containing a single uORF, scanning ribosomes bypass the inhibitory uORF due to the reduced efficiency of translation at initiation codons with a poor Kozak consensus sequence [16]. In both cases, the uORF bypass results in an increased number of ribosomes starting translation at the initiation codon of the main coding sequence, thereby increasing synthesis of that specific protein.

In this study, we demonstrate that *TMEFF2* translation is regulated by androgens. Androgen-regulation of *TMEFF2* translation requires the presence of the uORFs in the leader region of the *TMEFF2* mRNA and is dependent on eIF2α-P. Further, this effect is mediated by the AR since it is not observed when AR levels are reduced by RNAi or the AR antagonist bicalutamide, or in cell lines that do not express it. These results support a novel regulatory mechanism of androgen signaling in which uORF-containing mRNAs are translationally activated in response to eIF2α-P.

## Materials and Methods

### Cell Culture

LNCaP and 22RV1 cells were obtained from American Type Culture Collection and were maintained in RPMI 1640 (Gibco) supplemented with either 10% FBS (Gemini Bio-products) or 10% charcoal-stripped serum (Atlanta Biologicals). PC3 cells (ATCC) were obtained from Dr. D. Terrian (East Carolina University) and were also maintained in RPMI 1640. Mouse embryonic fibroblast expressing the wild type and mutant (Ser51 to Ala) eIF2 were obtained from Dr. R. Kaufman (Sanford/Burnham Medical Research Institute) and have been previously described [17]. These cells were grown in DMEM. Dihydrotestosterone, bicalutamide, and actinomycin D were purchased from Sigma-Aldrich.

### Constructs and Reporter Assays

PCR mutagenesis was used to mutate the start codons of the uORFs from AUG to GUG in the *TMEFF2* 5′ UTR. Primers used for mutagenesis are listed in table 1. The 5′ UTRs were inserted upstream of the Gaussia *luciferase* gene in the pCMV-GLuc vector (New England Biolabs). The TMEFF2-myc-his fusion construct has been previously described (11). For uORF analyses, cells were grown to 70–90% confluency in 6-well plates and transfected with 1.5 µg of each construct per well and the same amount of the pSeap-Control Vector II (BD Biosciences). Cells were transfected with Lipofectamine 2000 (Invitrogen) following the manufacturer’s protocol and using 4 µl/well of Lipofectamine reagent. Cells and supernatants were collected 24 hrs post-transfection, and luciferase activity was determined from the supernatants using the BioLux kit (New England Biolabs). Seap levels were measured using the Great Escape Seap Chemiluminescence Kit 2.0 (Clontech Laboratories). For both assays, luminescence was detected using a 20/20^n^ luminometer (Turner Biosystems).

**Table 1:** Primers used for plasmid construction or qRT-PCR detection.

Construct/gene name	Primer sequence
pTM1234-GLuc (wt)	5′-TAGGATCCCTCCACCCTGCCTCCTCG
	5′-TAACTAGTTCGTGCAACTCTGCAGCAG
pTMX234-GLuc	5′-GCTGCTGCCACAAGGAGGGAGC*
	5′-GCTCCCTCCTTGTGGCAGCAGC*
pTM1X34-GLuc	5′-GAGTTTCAGCAACACCCAGGGACT*
	5′-AGTCCCTGGGTGTTGCTGAAACTC*
pTM12X4-GLuc	5′-CCCGCGCACGATGTCGAGAG*
	5′-CTCTCGAGATCGTGCGCGGG*
pTM123X-GLuc	5′-GCTACTGAGCACCCCGCGGAC*
	5′-GTCCGCGGGGTGCTCAGTAGC*
*TMEFF2*	5′-TCTTGCAGGTGTGATGCTGG
	5′-GCTCCCTTTAGATTAACCTCG
*β-actin*	5′-GGACTTCGAGCAAGAGATGG
	5′-AGCACTGTGTTGGCGTACAG
*ATF4*	5′-TCAAACCTCATGGGTTCTCC
	5′-GTGTCATCCAACGTGGTCAG
Gaussia Luciferase	5′-GGAGGTGCTCAAAGAGATGG
	5′-TTGAACCCAGGAATCTCAGG

* The three-nucleotide mutation introduced is underlined.

For DHT-stimulated reporter assays, cells were hormone-starved for 48 hrs in phenol red-free medium containing 10% charcoal-stripped serum (CSS) prior to stimulation with DHT. Twenty-four hours after hormone removal the cells were transfected using Fugene HD transfection reagent (Promega) following the manufacturer’s recommendations. Briefly, 10 µg of each construct and 10 µg of pSeap2 were diluted in serum-free RPMI along with 30 µl Fugene HD reagent for a total volume of 500 µl. 100 µl of this transfection mix was added per well of a 6-well plate. The following day, DHT or ethanol vehicle were added to fresh CSS-RPMI and the cells were incubated for another 48 hrs prior to harvesting cells and supernatants. To measure Gaussia luciferase mRNA levels, RNA was isolated from the cells using the RNAqueous kit (Ambion) following the supplied protocol. cDNA was then synthesized using the iScript kit (BioRad Laboratories), and luciferase mRNA levels were measured by qRT-PCR using IQ SYBR Green Supermix and the IQ5 Real-Time PCR Detection System (BioRad Laboratories). Luciferase mRNA levels were normalized to β-*actin* and gene expression was analyzed using IQ5 Optical System Software (BioRad Laboratories). See table 1 for primers used for qRT-PCR.

### DHT Stimulation Time-course

Cells were hormone-starved in phenol red-free media containing 10% CSS overnight prior to stimulation with 10 nM DHT or DMSO control. Actinomycin D was used at a concentration of 5 nM. RNA was isolated from cells using the RNeasy Kit (Qiagen) and cDNA was synthesized using SuperScript II Reverse Transcriptase (Invitrogen). *TMEFF2* message levels were measured by qRT-PCR amplification using TaqMan *TMEFF2* primers (Hs00249367_m1, Applied Biosystems), and normalized to *GAPDH* message levels (amplified with Hs99999905_m1, Applied Biosystems).

### Androgen Receptor Knockdown

AR expression was reduced in 22RV1 cells using the ON-TARGET plus SMART pool for human AR (Thermo Scientific). Non-targeting siRNA was included as a control. Briefly, 5 µl of silencing RNAs and 7.5 µl of DharmaFECT Transfection Reagent (Thermo Scientific) were each separately diluted in 300 µl serum-free, phenol red-free RPMI. After 5 min incubation, the solutions were combined and incubated for another 20 min at room temperature, then added to an 85% confluent cell monolayer in a T-25 flask containing 2.4 ml of complete, phenol red-free RPMI.

### Immunoblotting

Cell lysates were prepared with RadioImmunoPrecipitation Assay (RIPA) buffer [25 mM Tris-HCl pH 7.6, 150 mM NaCl, 1% Trition X-100, 1% sodium deoxycholate, 0.1% SDS] supplemented with 0.1 mM β-glycerophosphate and 0.5 mM sodium orthovanadate and protease inhibitor cocktail (Sigma). Twenty µg of lysates were separated on Mini-protean TGX gels (BioRad) and transferred to PVDF membranes. These were then blocked for 30 min in 5% NFDM diluted in TBS-T and incubated with the primary antibody overnight at 4°C. Incubations with a 1∶10,000 dilution of HRP-conjugated secondary antibody (Santa Cruz Biotechnology) were for 1 hr at room temperature. Detection was carried out using SuperSignal West Pico Chemiluminescent Substrate (Thermo Scientific) for 5 min. In some cases, blots were stripped with Restore PLUS Western Blot Stripping Buffer (Thermo Scientific) following the manufacturer’s recommendations. Antibodies against TMEFF2, PSA, and eIF2α-P (Ser51) were from Abcam, eIF2α and CREB2/ATF4 antibodies were purchased from Santa Cruz Biotechnology, and P-eIF2α (Ser51) antibodies were from Cell Signaling Technology.

### Polysome Analysis

Cell monolayers were washed once with cold 1X PBS and scraped with lysis buffer [100 mmol/L KCl, 10 mmol/L HEPES (pH 7.4), 0.5% NP40, 5 mmol/L MgCl_2_, 100 µg/ml cycloheximide], and incubated on ice for 10 mins, followed by a 5 min spin at 10,000 rpm at 4°C to pellet cellular debris. Equal protein concentrations of cytoplasmic extracts (1.8 mg) were then overlaid onto a linear sucrose gradient [15–45% (w/v) 10 mmol/L HEPES (pH 7.4), 100 mmol/L KCl, 5 mmol/L MgCl_2_] and centrifuged at 35,000 rpm for 2 hrs at 4°C in an SW41-Ti rotor without the brake. Using an ISCO UA-6, fractions were collected with continuous UV monitoring at 254 nm. RNA was isolated from fractions using Trizol reagent (Ambion). Twenty-five micrograms of RNA were then used for cDNA synthesis using an iScript kit (BioRad Laboratories). A sample of 0.1 µg of each cDNA preparation was used to amplify *TMEFF2*, *ATF4*, and *β-actin* by PCR using the Platinum Taq HiFi DNA Polymerase system (Invitrogen). PCR products were then visualized on a 1% agarose gel and analyzed using the public domain NIH Image J program (developed at the U.S. National Institutes of Health and available on the Internet at http://rsb.info.nih.gov/nih-image/).

### Immunoflourescence

Cells were plated at a density of 10,000 cells/well in Lab-Tek 8 well chambered slides (Nalge Nunc International) and incubated overnight in a humidified 37°C incubator with 5% CO_2_. Cells were washed in PBS and fixed in 4% paraformaldehyde for 10 minutes, washed in PBS and permeabilized with 0.1% Triton-X100 in PBS and blocked with 5% normal goat serum in 0.1% PBST. Samples were incubated for 1 hour with the indicated primary antibodies. The corresponding secondary antibodies were added at a 1∶500 dilution in 5% goat serum in PBS-T (0.1% Tween 20): goat anti-rabbit IgG-AF488 (Invitrogen) and goat anti-mouse IgG-AF488 (Invitrogen). Cells were washed 3 times in PBS-T (0.1% Tween 20). Slides were air-dried and mounted in medium containing DAPI.

### Subcellular Fractionation

Extracts from cells grown in the presence of 10 nM DHT, or DMSO as control, were fractionated into cytosolic, membranous, nuclear and cytoskeletal fractions using the Subcellular Proteome Extraction kit (Calbiochem).

### Statistical Analysis

Data are expressed as mean ± SD. Differences were analyzed using paired, two-tailed t-tests. P values ≤0.05 (*) or ≤0.01 (**) were considered significant.

## Results

### The *TMEFF2* 5′-UTR Contains Several uORFs that Block Translation of the TMEFF2 Protein

**Figure 1:**
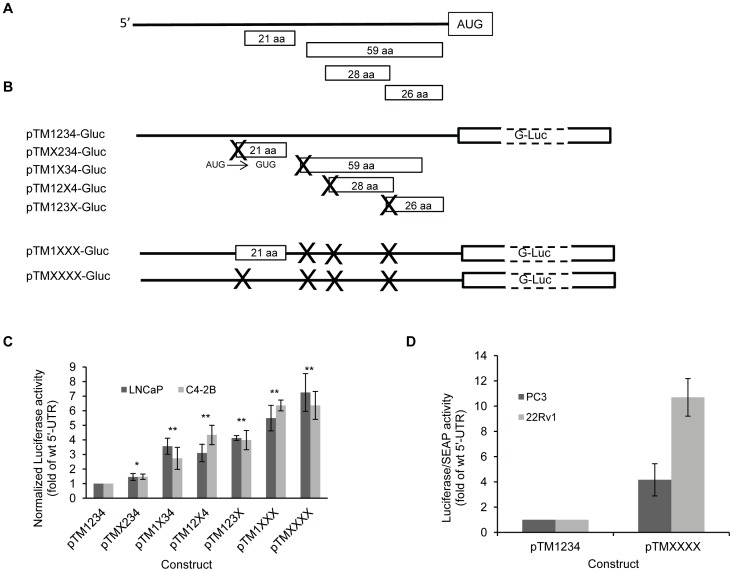
The uORFs in the 5′-UTR of *TMEFF2* inhibit translation of the main coding region. **A)** Schematic representation of 394 nt upstream of the *TMEFF2* main coding region and relative localization of four potential uORFs (aa = amino acid) within the 5′-UTR. **B)** Schematic representation of the TMEFF2-Gaussia Luciferase reporter and mutant constructs. The X indicates mutation of the AUG to a GUG to prevent translation of the mutated uORFs. Single and multiple mutations were introduced. **C)** luciferase activity demonstrated by the pTM1234-Gluc and the different mutant constructs in LNCaP and C4-2 cells. **D)** Luciferase activity demonstrated by the pTM1234-Gluc and the construct with all the uORFs mutated (pTMXXXX-Gluc) in PC3 and 22Rv1 cells. In **C)** and **D)** luciferase activity was measured in the supernatant and calculated by first normalizing to mRNA levels for each construct and then to the luciferase activity demonstrated by the pTM1234-Gluc reporter construct, which does not have mutations in the uORFs, considered arbitrarily as 1. Data shown are mean ± S.D. of at least two independent experiments with multiple replicates. *, *p*<0.05, and **, *p*<0.01.

The 5′ UTR of the *TMEFF2* mRNA contains several potential uORFs (Figure 1A) that, if translated would potentially block translation of the *TMEFF2* main coding sequence, and therefore contribute to the regulation of *TMEFF2* expression. To investigate the role of the uORFs in regulating TMEFF2 protein expression, we asked whether blocking translation of the uORFs would affect translation of the TMEFF2 protein in human prostate cancer cell lines. A TMEFF2-Gaussia Luciferase (GLuc) reporter was generated for this purpose by cloning the *TMEFF2* 5′-UTR, including four uORFs, upstream of the GLuc sequences (pTM1234-Gluc; Figure 1B). The *TMEFF2*-Gluc fusion was placed under control of the CMV promoter. The regulatory contribution of the uORFs to *TMEFF2* translation was evaluated by mutating the start codons (AUGs) of the four potential uORFs (AUG to GUG, Figure 1B) and determining their effect on GLuc expression in the androgen-dependent prostate cancer cell line LNCaP and its bone-metastatic, androgen-independent derivative C4-2B cell line. A six- to seven-fold increase in Luciferase expression was observed when the AUGs from all four uORFs were mutated (pTMXXXX-Gluc; Figure 1C). Single mutations on the AUGs of the second, third or fourth uORFs promoted a 3–4 fold increase in GLuc expression, while mutating the AUG of the first uORF had a very small effect, suggesting a minimal role, if any, in regulation of TMEFF2 expression. Accordingly, combined mutations of uORFs 2, 3, and 4 resulted in a five- to six-fold increase in Luciferase expression of the reporter, similar to that observed when all four uORFs were mutated (Figure 1C). Similar results were obtained in other androgen-responsive (22Rv1) and -independent (PC3) prostate cancer cell lines. Mutation of the uORFs resulted in increased Luciferase expression of the GLuc reporter, albeit at variable fold induction (Figure 1D). In the constructs used for these experiments, expression of the fusion gene was directed by the CMV promoter, and the luciferase activity was normalized to mRNA levels. Altogether, these results suggest that the uORFs in the 5′- UTR of *TMEFF2* mRNA function synergistically to repress translation of the main downstream ORF.

### Translation of a TMEFF2-Luciferase Reporter is Regulated by Androgens through a Mechanism that Requires the Presence of the uORFs in the mRNA Leader Region

**Figure 2:**
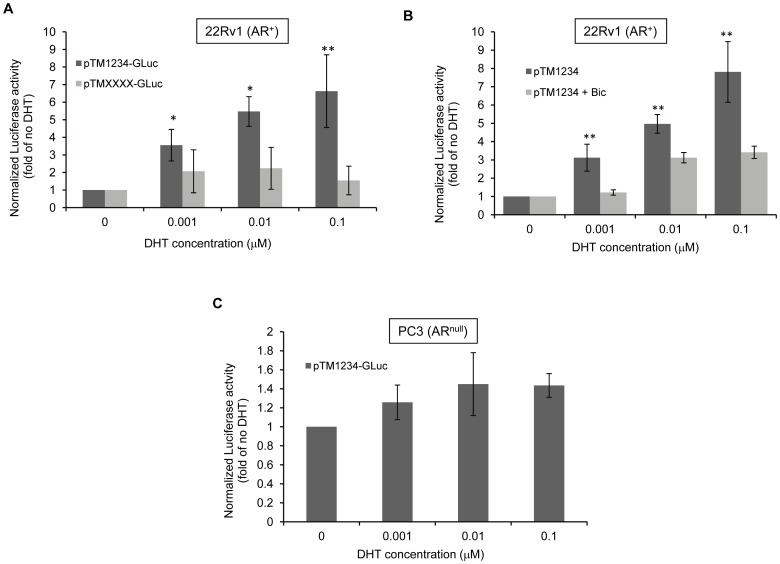
DHT promotes AR-mediated increased translation of the TMEFF2-GLuc fusion protein in 22Rv1 prostate cancer cells. **A)** Luciferase activity demonstrated by the pTM1234-Gluc and the pTMXXXX-Gluc mutant construct in 22Rv1 cells in the presence of different concentrations of DHT. **B)** Luciferase activity demonstrated by the pTM1234-Gluc construct in 22Rv1 cells in the presence of different concentrations of DHT and DHT +20 µM bicalutamide (bic), an AR agonist. **C)** Luciferase activity demonstrated by the pTM1234-Gluc construct in PC3 cells in the presence of different concentrations of DHT. For all these experiments cells were hormone-starved in phenol red-free media containing charcoal stripped serum. Luciferase activity was normalized to mRNA levels for each construct and then to the luciferase activity demonstrated by each one of the constructs expressed in cells grown in the absence of DHT, which was set to 1. Data shown are mean ± S.D. of at least three independent experiments with multiple replicates. *, *p*<0.05, and **, *p*<0.01.

Transcription of the *tmeff2* gene is regulated by androgens [8]. However, it has been suggested that androgens also affect *TMEFF2* expression at the post-transcriptional level [10], prompting us to examine whether *TMEFF2* translation was affected by androgens. 22Rv1 cells were selected for these experiments since: i) they have been shown to be a valuable model for AR-mediated reporter gene assays [18], ii) they demonstrated the highest fold increase in Luciferase reporter gene expression when the uORFs were mutated (see Figure 1D), and iii) they express detectable levels of endogenous TMEFF2 protein. 22Rv1 cells were transfected with the pTM1234-Gluc reporter, grown in phenol red-free media supplemented with charcoal-stripped (CS) FBS – to remove steroid hormones- and treated with different concentrations of dihydrotestosterone (DHT). Luciferase activity was measured from the supernatants and normalized to mRNA levels. Addition of DHT increased Luciferase expression in a dose-dependent manner (Figure 2A), indicating that androgens stimulate the translation of the main ORF. Importantly, this effect was observed at DHT concentrations within the physiological levels found in human serum [19]. Luciferase activity from cells carrying the pTMXXXX-Gluc reporter construct, in which the AUGs from all four uORFs were mutated, did not change in response to DHT, although, as expected, was much higher (Figure 2A). These results indicate that translation of the main ORF downstream of the *TMEFF2* 5′- UTR is regulated by androgens in an uORF-dependent manner.

To determine whether the DHT effect on translation is mediated by the AR, the experiments described above were repeated in the presence of bicalutamide to block AR activation. Addition of this drug reduced the DHT-mediated induction of the pTM1234-Gluc reporter Luciferase expression to near basal levels, although a small two- to three-fold induction could be observed at 10 nM DHT (Figure 2B). These results indicate that the effect of DHT on translation of the reporter construct requires AR signaling. In agreement with these results, we did not observe DHT-induced translation of the pTM1234-Gluc reporter in PC3 prostate cancer cells that do not express the AR (Figure 2C). Altogether, these results indicate that DHT-induced translation of *TMEFF2* requires activation of the AR and is mediated by the uORFs in the leader region of the mRNA.

### Translation of the Endogenous TMEFF2 Protein is Regulated by Androgens

**Figure 3:**
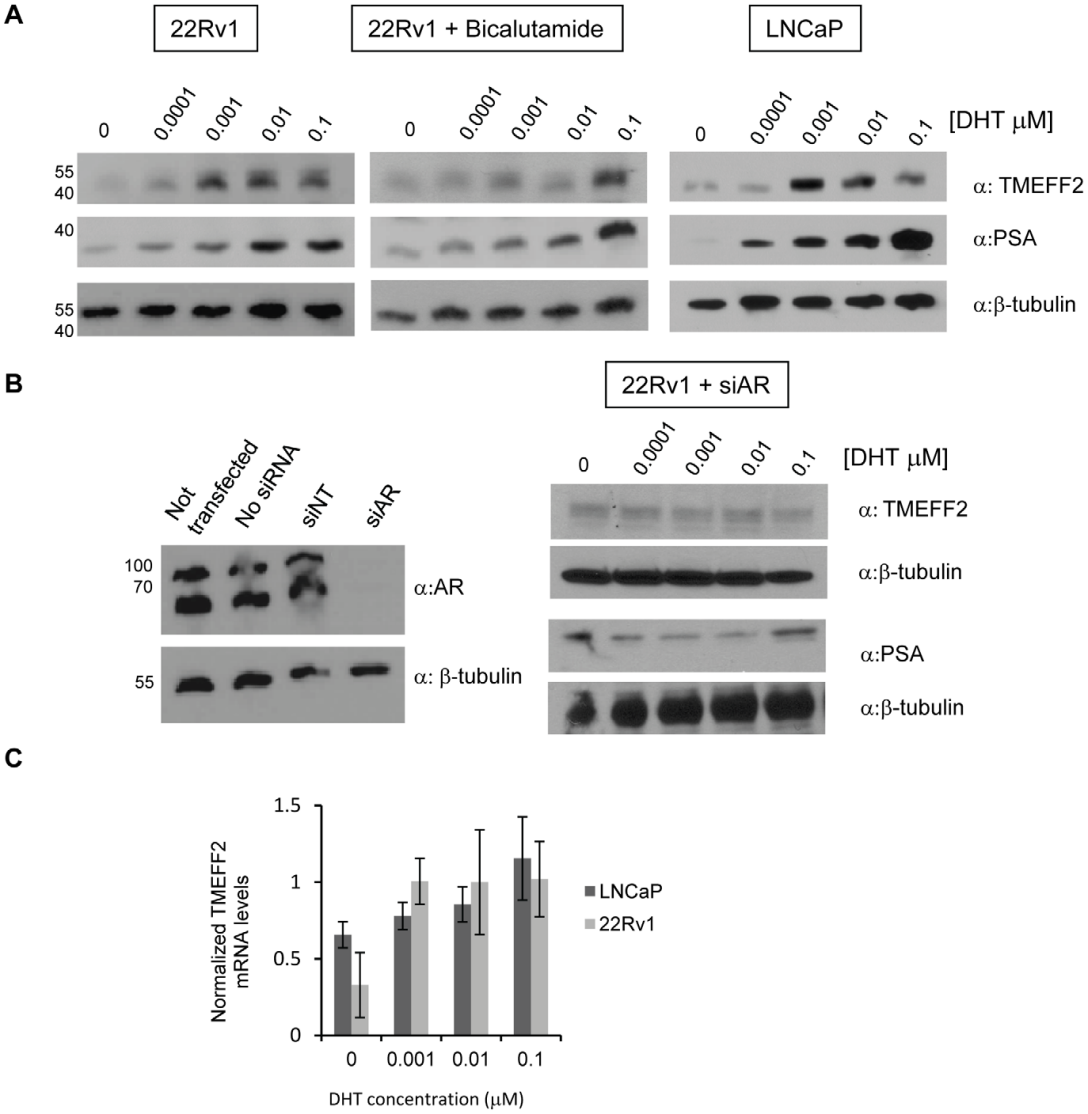
DHT promotes AR-mediated increased expression of the endogenous TMEFF2 protein in prostate cancer cells. **A)** Representative western blots indicating an increase in TMEFF2 expression in response to DHT addition in 22Rv1 and LNCaP cells (α = antibody). Simultaneous addition of 20 µM bicalutamide to 22Rv1 cells (middle panel) prevented the increase in TMEFF2 expression observed at physiological concentration of DHT. PSA was used as positive control since its expression is induced by androgen in an AR-dependent manner. β-tubulin was used as a loading control. **B)** Western blot indicating effective knock down of the two forms of the AR in 22Rv1 cells using siRNA (right). Representative western blot indicating that addition of DHT has no effect on the expression of TMEFF2 in cells in which AR levels were reduced by RNAi. PSA was used as control and, as expected, its expression was not affected by DHT in the AR-siRNA treated cells. β-tubulin was used as a loading control. **C)** Changes in *TMEFF2* mRNA levels in LNCaP and 22Rv1 cell lines in response to DHT as measured by qRT-PCR. Values were normalized to β-tubulin mRNA. Each experiment was repeated at least three times and, for the representative images presented, the membranes were stripped and re-probed with the different antibodies or the same samples were re-run in a different gel. β-tubulin was used as a loading control each time the samples were run.

Changes in the expression of the endogenous TMEFF2 protein in response to androgens were also analyzed. For this purpose, 22Rv1 cells were grown in phenol red-free media supplemented with CS-FBS treated with different concentrations of DHT, and lysates analyzed for TMEFF2 expression by western blotting. In the absence of androgens, expression of TMEFF2 was barely detectable. However, addition of DHT increased TMEFF2 expression (Figure 3A), and resulted in the highest levels within the range of physiological DHT concentrations. DHT-induced expression of endogenous TMEFF2 was also observed in the androgen-responsive prostate cancer LNCaP cells (Figure 3A). The expression of prostate specific androgen (PSA), an AR target used as control for androgen transcriptional activity, was enhanced by the addition of DHT (Figure 3A). Treatment of the cells with bicalutamide notably inhibited DHT-induced TMEFF2 and PSA expression that was only observed at the highest concentrations of DHT (Figure 3A). Inhibition of DHT-induced TMEFF2 expression was also achieved after knocking down expression of the AR using siRNA (Figure 3B). Altogether, these results indicate that the expression of the endogenous TMEFF2 protein is regulated by AR signaling.

**Figure 4:**
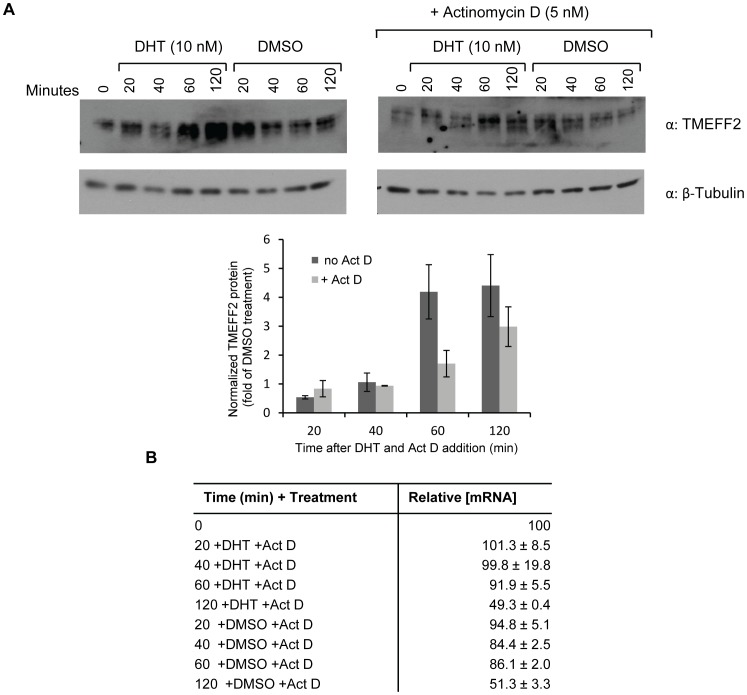
DHT promotes increased transcription and translation of the endogenous *tmeff2* gene. **A)** Representative western blot demonstrating the TMEFF2 protein at the indicated time points after addition of 10 nM DHT in the presence or absence of 5 nM actinomycin D (Act D) to block transcription. Either DMSO or DHT and actinomycin D were added simultaneously. Quantitation of the blots is presented below the western blot. The band intensities of the samples treated with DHT were normalized first to β-tubulin and then to the values obtained for the DMSO samples at the corresponding time points. **B)** Changes in *TMEFF2* mRNA levels in 22Rv1 cells in response to DHT or DMSO and actinomycin D treatment as measured by qRT-PCR. Values were normalized to *GAPDH*. Data shown are representative of two independent experiments.

We observed an increase in *TMEFF2* mRNA levels in response to DHT as measured by qRT-PCR (Figure 3C), consistent with a previous report indicating that androgens stimulate *tmeff2* transcription [8]. However, the modest increase in mRNA levels does not likely explain the observed DHT-mediated increase in protein expression. To rule out the possibility that the increase in endogenous TMEFF2 protein observed in response to DHT was solely due to increased transcription, we used actinomycin D to block transcription and assessed the levels of TMEFF2 protein and mRNA in 22Rv1 cells at different time points after the addition of DHT, to induce *tmeff2* expression, and/or actinomycin D. TMEFF2 protein levels increased dramatically 60–120 mins after the addition of DHT, but not DMSO, the vehicle control (Figure 4A). Importantly, in the presence of actinomycin D, addition of DHT still promoted an increase in TMEFF2 protein expression, albeit at a lower level than observed in the absence of the transcription inhibitor. As indicated by analysis of the *TMEFF2* mRNA levels by qRT-PCR, addition of actinomycin D inhibited basal and DHT-induced *tmeff2* transcription (Figure 4B). Addition of DHT alone promoted a slight initial increase in transcription (1.2-fold after 20 mins) that progressively declined to basal levels after 60 mins of treatment (not shown). Therefore, these results reveal that androgens regulate not only transcription of the *tmeff2* gene but also translation of the endogenous *TMEFF2* mRNA, supporting the results presented above using a TMEFF2-Luciferase reporter.

Other proteins including β-catenin and occludin translocate to the nucleus or regions of cellular contact after androgen treatment. However, our results indicated that androgen treatment did not alter the subcellular localization of TMEFF2 (Figure S1).

### Androgen Signaling Promotes eIF2α Phosphorylation

**Figure 5:**
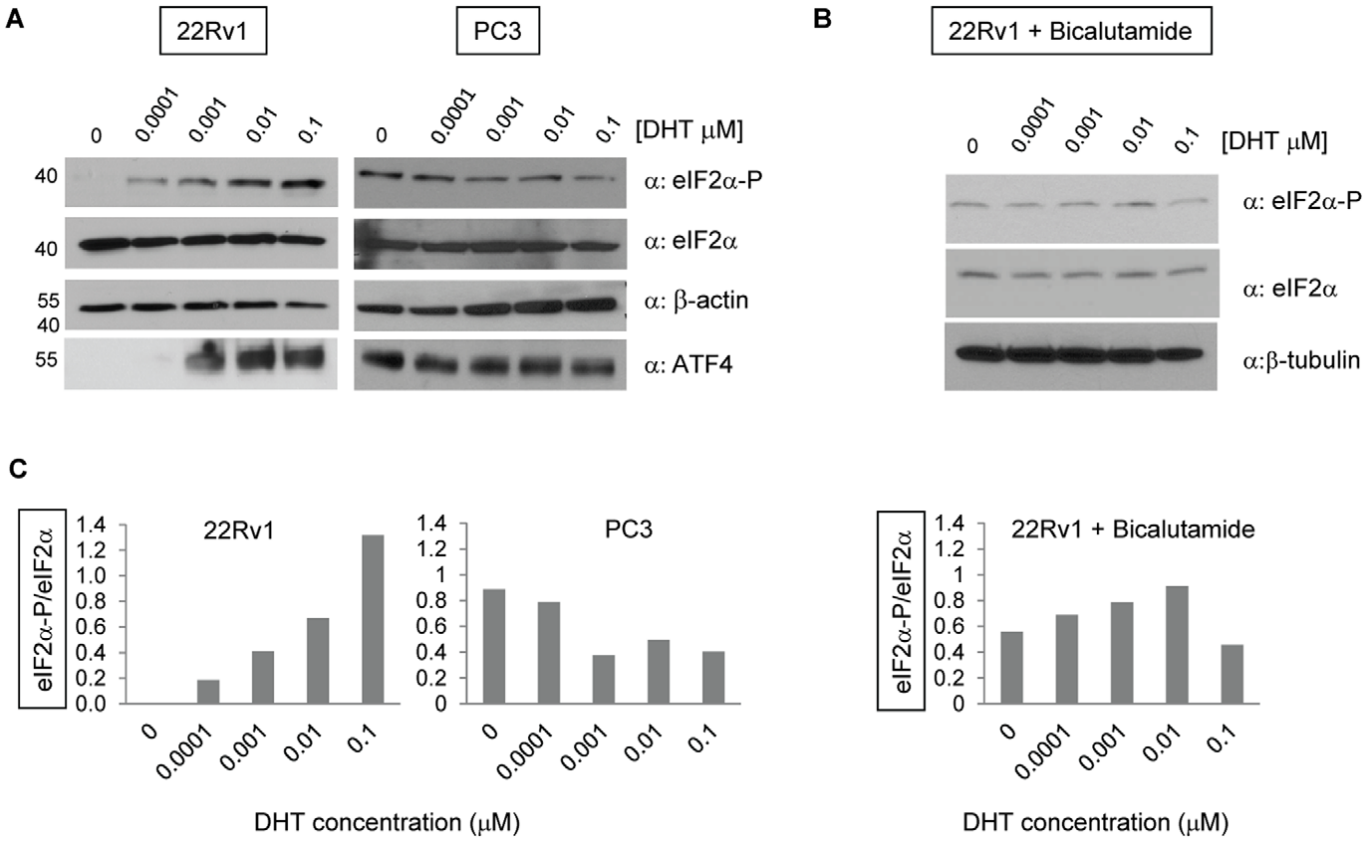
DHT induces phosphorylation of eIF2α in an AR-dependent manner. **A)** Representative western blots indicating an increase in eIF2α-P in response to DHT addition in 22Rv1 cells. This effect was abrogated in PC3 cells, which do not express AR (right). ATF4 protein levels were measured as a positive control since it is induced by eIF2α-P. β-actin was used as a loading control. **B)** Addition of 20 µM bicalutamide to 22Rv1 cells prevented the increase in eIF2α-P observed after addition of DHT. β-tubulin or β-actin were used as loading controls. **C)** Densitometry of the western blot bands from A) and B). The ratio of eIF2α-P to total eIF2α for each specific cell line/treatment is graphed to determine the changes in eIF2α-P promoted by DHT treatment.

Phosphorylation of eIF2α reduces global translation but also provides a mechanism that selectively enhances translation of uORF-containing mRNAs [13], [20]. We therefore hypothesized that DHT promotes endogenous *TMEFF2* translation through phosphorylation of eIF2α. The effect of DHT on eIF2α phosphorylation was examined by western blot analysis in prostate cancer 22Rv1 and PC3 cells grown in phenol red-free media supplemented with CS-FBS and treated with different concentrations of DHT. Increased levels of eIF2α-P were clearly detected, in a dose-dependent manner, in lysates from DHT-treated 22Rv1 cells but not in lysates from DHT-treated AR-null PC3 cells (Figure 5A), indicating that androgens promote eIF2α-P and that this effect is dependent on the presence of a functional AR. In agreement with these results, pretreatment of the 22Rv1 cells with the AR antagonist bicalutamide prevented DHT-mediated increases in eIF2α phosphorylation (Figure 5B). DHT addition also promoted an increase in the expression of the ATF4 protein, a transcription factor regulated by eIF2α phosphorylation (Figure 5A).

### Conditions that Promote eIF2α-P Result in Increased TMEFF2 Translation

**Figure 6:**
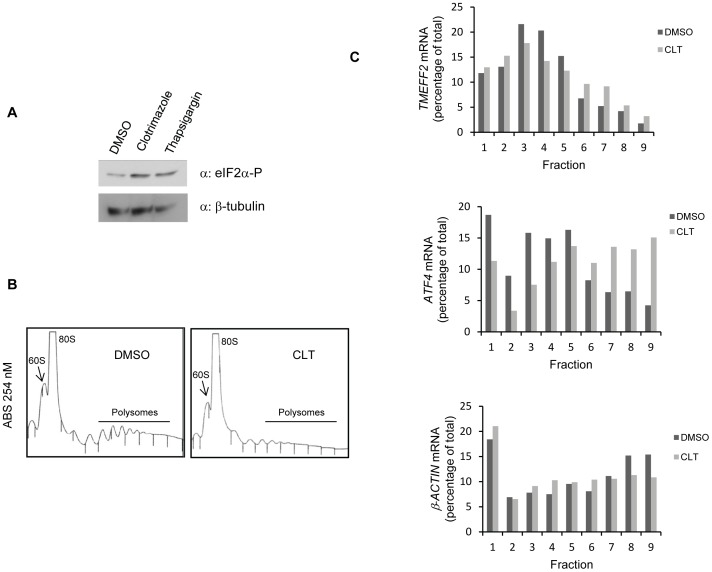
*TMEFF2* mRNA associates with heavier polysomes in response to ER stress. **A)** Western blot indicating phosphorylation of eIF2α in response to ER-stress inducing agents in 22Rv1 cells. **B)** 22Rv1 cells were exposed to 15 µM clotrimazole (CLT) or vehicle control (DMSO) for 1 hr and lysates subjected to polysome analysis. A representative example of one of the three independent polysome profiles obtained is shown. **C)** Total RNA was prepared from the fractions, and the percentage of *TMEFF2*, *ATF4*, and *β-actin* mRNAs present in each fraction were determined by qRT-PCR. Results from one of three independent experiments are shown.

In order to determine whether eIF2α phosphorylation is sufficient to regulate translation of *TMEFF2*, 22Rv1 cells were treated with clotrimazole, a drug that causes depletion of intracellular Ca^2+^ stores, resulting in activation of the PKR kinase and subsequent eIF2α-P, and the effect on *TMEFF2* translation efficiency was analyzed using polysome analysis. Western blot analyses indicated that the clotrimazole treatment resulted in increased phosphorylation of eIF2α in 22Rv1 cells (Figure 6A). As previously described [21], [22], clotrimazole treatment resulted in reduced polysomes along with an increase in monosomes indicating inhibition of translation initiation (Figure 6B). Under these conditions, we observed a shift of the *TMEFF2* mRNA towards the heavier polysomal fractions when compared to the DMSO-treated controls (Figures 6B and 6C), suggesting that clotrimazole treatment increased translation of the *TMEFF2* mRNA. However, the shift was small, likely reflecting the presence of multiple uORFs and a complex translational regulatory mechanism. Similar results were observed in other cell lines (data not shown). Confirming these results, clotrimazole treatment of cells containing the pTM1234-Gluc reporter resulted in a significant increase in luciferase activity (data not shown). In addition, a shift to the heavier polysomal fractions was also observed for the uORF-containing *ATF4* mRNA, known to be preferentially translated upon eIF2α-P (Figures 6B and 6C; [23]). Taken together, these results demonstrate that eIF2α phosphorylation, independent of the causative stimulus, is sufficient to enhance translation of *TMEFF2*.

### eIF2α Phosphorylation is Essential for Increased Translation of a TMEFF2-GLuc Reporter in Response to ER Stress

**Figure 7:**
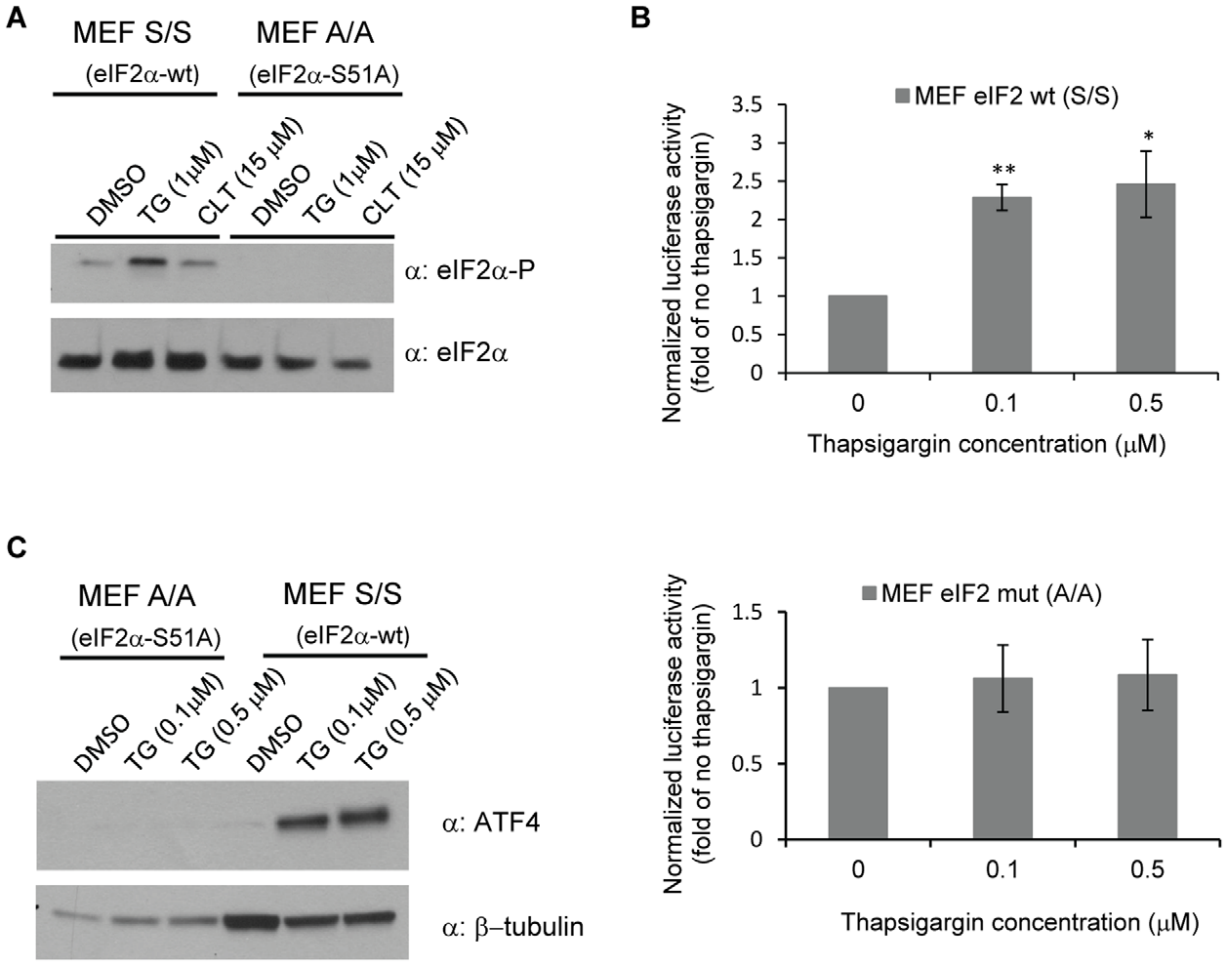
Phosphorylation of eIF2α is required for increased *TMEFF2* translation in response to ER stress. **A)** Mouse embryonic fibroblast (MEF) cells expressing the wild type (S/S) or mutant (Ser51 to Ala; A/A) eIF2 proteins were treated with the indicated concentrations of clotrimazole (CLT) or thapsigargin (TG) and protein lysates from these cells were analyzed by western blot to determine the extent of eIF2α-P. Total eIF2α was used as loading control. **B)** Luciferase activity demonstrated by the pTM1234-Gluc reporter construct in wt (S/S) or mutant (A/A) MEFs in the absence or presence of the indicated concentrations of thapsigargin. Luciferase activity was normalized to the luciferase activity of the reporter expressed in cells grown in the absence of thapsigargin, which was set to 1. Data shown are mean ± S.D. of at least three independent experiments with multiple replicates. *, *p*<0.05, and **, *p*<0.01. **C)** Western blot analysis demonstrating increased ATF4 expression as a result of thapsigargin treatment in MEF cells carrying the wt form of the eIF2α subunit (MEF S/S) but not the mutant form mutant (MEF A/A). β-tubulin was used as a loading control.

To further investigate the role of eIF2α phosphorylation on translation of *TMEFF2*, mouse embryonic fibroblasts (MEFs) expressing either wild-type eIF2α (S/S) or an eIF2α S51A (A/A) mutant form, carrying a Ser to Ala mutation that prevents phosphorylation of eIF2α, were transfected with the pTM1234-Gluc reporter and expression of the reporter analyzed after addition of thapsigargin, a drug that disrupts ER calcium homeostasis leading to kinase activation and eIF2α phosphorylation. This experimental system was tested by treating the cells with clotrimazole or thapsigargin and determining that both drugs effectively promoted eIF2α-P in cells carrying the wild-type but not the S51A (A/A) mutant eIF2α (Figure 7A). Since thapsigargin demonstrated a more robust response, it was chosen for subsequent experiments. MEFs expressing the pTM1234-Gluc reporter were grown in phenol red-free media supplemented with CS-FBS in the absence or presence of different concentrations of thapsigargin. Increased luciferase activity from the pTM1234-Gluc reporter was detected in response to 0.1 and 0.5 µM thapsigargin in cells carrying the wild-type eIF2α but not in the eIF2α S51A mutant cells (Figure 7B). Higher concentrations of thapsigargin did not result in higher luciferase activity and reached a toxic level (data not shown). Similarly, thapsigargin was able to increase ATF4 protein levels used as a positive control for eIF2α phosphorylation, only in the cells carrying the wild-type eIF2α allele (Figure 7C). These results confirm that phosphorylation of eIF2α is required to induce translation of the TMEFF2-Gluc reporter observed in response to ER stress.

## Discussion

Changes in the expression of TMEFF2 protein have been documented in prostate cancer, and a potential role for TMEFF2 in this disease has been proposed [7]–[11]. However, the molecular mechanisms leading to deregulation of TMEFF2 expression in prostate cancer are not known. The present study reveals a novel role for androgens in the regulation of *TMEFF2* translation that could account for the changes in TMEFF2 expression observed in prostate cancer. We demonstrate that androgen signaling promotes increased *TMEFF2* mRNA translation through a mechanism that involves phosphorylation of eIF2α and is dependent on the presence of uORFs in the 5′-UTR of the *TMEFF2* mRNA.

The main androgen, testosterone, and its metabolite, DHT, largely mediate their effect through binding to the AR, a ligand-inducible transcription factor. The bound AR can then recognize and bind specific androgen response elements in the promoter regions of target genes, causing activation or repression of transcription, leading to subsequent changes in protein synthesis [24]. However, post-transcriptional regulation of gene expression by androgens has also been documented, affecting mRNA stability, protein localization, polyA-tail length, and translation efficiency [25]–[27]. The effect of androgens on translation is mediated through several mechanisms including changes in miRNA expression or processing [28]–[30], phosphorylation of translation factors like eEF2 [31], or mTOR activation-mediated phosphorylation of p70S6 and 4E-BP1 [32]. The results presented here indicate that physiological concentrations of DHT promote eIF2α phosphorylation in prostate cancer cell lines to influence translation.

Phosphorylation of eIF2α ultimately leads to inhibition of global translation initiation along with stimulation of translation of specific mRNAs, i.e. uORF-containing mRNAs. These changes facilitate cellular adaptation to the stress conditions promoting either survival or initiation of apoptosis [33]. Using TMEFF2-Luciferase reporter constructs in which the 5′-UTR of *TMEFF2* was fused to the Gaussia *luciferase* gene, we demonstrated that, in addition to phosphorylating eIF2α, DHT also promotes translation of TMEFF2. Importantly, this effect requires the presence of uORFs in the 5′-UTR of the *TMEFF2* mRNA, as DHT showed no effect on translation of a TMEFF2-Luciferase reporter in which the AUGs for each of the uORFs were mutated. Supporting these results, translation of the TMEFF2 endogenous protein and the uORF-containing ATF4 transcriptional regulator, (known to be regulated by eIF2α-P) were also increased in response to DHT stimulation. Moreover, as previously demonstrated for *ATF4* [34], polysome analysis revealed that translation efficiency of *TMEFF2* increased in response to clotrimazole, a translation inhibitor that promotes ER stress and subsequent eIF2α-P. In addition, the DHT effect on *TMEFF2* translation was not observed in cells carrying a non-phosphorylatable form of eIF2α. These results suggest that the effect of DHT in TMEFF2 protein expression was mediated by eIF2α-P. We also demonstrated that the DHT effect on TMEFF2 protein expression required the presence of a functional AR, but was partially independent of transcriptional activity. To our knowledge, this is the first report demonstrating a transcription-independent effect of DHT on translation of uORF-containing mRNAs through phosphorylation of eIF2α.

Phosphorylation of eIF2α in response to different forms of stress is mediated by one of the following four kinases [35]: protein kinase R (PKR), heme-regulated eIF2α kinase (HRI), protein kinase R-like kinase (PERK), or GCN2 (general control nonderepressible-2). Several different kinases might be responsible for eIF2α phosphorylation in response to DHT. Androgens regulate transcription of many genes, including some of those involved in amino acid metabolism [26], [36], [37], and it is therefore possible that DHT-induced eIF2α phosphorylation results from GCN2 activation driven by metabolic changes. However, under our experimental conditions, DHT stimulated translation of *TMEFF2* in the presence of actinomycin D, suggesting that the DHT-induced phosphorylation of eIF2α is, at least partly non-genomic. While this manuscript was in preparation, Dai and collaborators [38] reported increased apoptosis of human liver cells in response to DHT administration through a PKR-eIF2α-P dependent mechanism. This effect was sensitive to the AR antagonist flutamide and it seemed to involve CHOP (GADD153) activation. These results suggest that DHT functions by activating PKR, however, the experiments were performed in the presence of 100 nM DHT, a concentration much higher than physiological levels (1–10 nM). In this regard, studies in mice indicate that excess androgens can promote oxidative stress [39]–[42], which can induce PKR activity [43] and phosphorylation of eIF2α. In cell lines, addition of physiological levels of DHT can promote increased cytosolic calcium (Ca^2+^), which can promote PKR activation either directly or indirectly by increasing oxidative stress [44]–[46]. Therefore, DHT could indirectly promote PKR activation through its ability to promote accumulation of intracellular calcium, a well-known transcription independent, non-genomic effect of androgen.

TMEFF2 is mainly expressed in the adult brain and prostate where it plays a role as a tumor suppressor [7]–[9], [11], [47]. In these two tissues, androgens/AR signaling are known to regulate the frequency and extent of male typical behaviors [48] and the development, function, and homeostasis of the prostate as well as prostate cancer initiation and progression [49], respectively. Interestingly, basal TMEFF2 protein expression has been reported in other tissues such as mouse white adipose tissue [50] and rat heart and kidney ([51]; see also human data available in the gene expression Atlas), in which the role of androgen/AR signaling is not clear. However, in these tissues, the presence of the AR has been thoroughly described and some effects of androgens have been reported [52]–[56]. Therefore, although not yet explored, it is possible that expression of TMEFF2 in these tissues is also regulated by androgen signaling.

What is the role of AR-mediated phosphorylation of eIF2α in prostate cancer? A correlation between translation initiation and prostate cancer can be postulated from the observation that several translation initiation factors are overexpressed or activated in this disease [57]. For example, eIF3h and eIF4E are frequently overexpressed in advanced prostate cancers, together with increased phosphorylation of eIF4E and eIF4E-BP1, which support increased translation [58]. However, the role of eIF2α-P in prostate or other cancers is not clear. While expression of a non-phosphorylatable form of eIF2α or overexpression of a dominant-negative form of PKR have been shown to inhibit apoptosis and cause malignant transformation, other reports indicated that reduced PKR levels correlated with less aggressive tumors [58]. In general, it has been suggested that the role of eIF2α-P is dependent on the stage and grade of the disease, occurring at the earliest stages as a response to stress, while downregulation of eIF2α-P will occur later on as a result of the selective pressure imposed by the tumor’s cell need to proliferate [57]. Androgen signaling is critical for the progression of prostate cancer, and changes in the sensitivity to androgen signaling after prostate cancer regression drives the cancer to the advanced stages. It is possible that early in prostate cancer, or upon recurrence, androgen signaling triggers PKR activation and subsequent eIF2α-P, leading to anti-proliferative effects, apoptosis, and the activation of tumor suppressor mechanisms. In this respect, it has been reported that the antiproliferative and pro-apoptotic effects of PTEN, a tumor suppressor protein frequently mutated in prostate cancer, require activation of the PKR-eIF2α-P pathway [59]. Persistent AR signaling along with dowregulation of eIF2α-P may lead to AR-mediated transcriptional events and tumor progression. Interestingly, while the relative contribution of androgen-dependent transcriptional and translational mechanisms to the regulation of TMEFF2 expression cannot be accurately determined from our studies, previous prostate cancer xenograft data demonstrated that TMEFF2 protein, but not mRNA, levels were increased in recurrent tumors and in tumors obtained from 6-day castrate mice treated with testosterone [10], and correlate with the expression of androgen receptor [60]. These results suggest that translational control by AR signaling may play an important role on TMEFF2 regulation during the transition to castration resistant prostate cancer.

In summary, our findings reveal a novel role for AR signaling as a translational regulator of TMEFF2 through phosphorylation of eIF2α. We have previously demonstrated that the tumor suppressor ability of TMEFF2 partly correlates with its ability to interact with SARDH and modulate the levels of sarcosine [11]. SARDH and GNMT, the enzymes that catalyze the forward and reverse conversion of sarcosine into glycine, are under transcriptional control mediated by the androgen receptor. Treatment of prostate cancer cell lines with androgens resulted in an increase in GNMT expression and a simultaneous decrease in SARDH levels [12]. Since *tmeff2* is both transcriptionally [8] and translationally regulated by androgens, these observations potentially link androgens with the regulation of sarcosine metabolism and changes in TMEFF2 expression, and may suggest a model by which in the early stages of tumorigenesis the increased expression of TMEFF2 in response to androgens is a cellular response to overcome tumorigenesis. Persistent AR signaling leads to an increase in GNMT and a simultaneous decrease in SARDH expression that overcome the effect of increased TMEFF2 levels. Therefore, TMEFF2 upregulation in response to AR signaling may initially serve as a barrier for malignant progression of prostate cancer.

Genomic, proteomic and metabolomic studies have implicated androgens/AR signaling in regulating metabolic processes in prostate cancer. Relevant to our studies, recent data from the Sreekumar laboratory uncovered a role for androgens in activating amino acid metabolism and methylation in prostate cancer cells [61]. Since sarcosine, SARDH, GNMT and TMEFF2, are directly involved in 1-carbon metabolism and therefore S-adenosylmethionine utilization, it is possible that AR exerts its effect on methylation at least in part by modulating the enzymes involved in the sarcosine-related steps in the pathway. Interestingly, AR expression, activity and function are methylation-dependent [62]–[65], suggesting a potential feed-forward mechanism by which AR increases *TMEFF2* expression leading to changes in the methylation potential of the cell that consequently affects AR function. A potential direct effect of TMEFF2 on AR function is currently under investigation.

## Supporting Information

Figure S1 **DHT does not alter the subcellular localization of TMEFF2.** The potential effect of DHT on TMEFF2 localization was examined by subcellular fractionation (A) or immunofluorescence (B). **A)** 22Rv1 cells were treated with DHT or the vehicle control and subcellular fractions separated and analyzed by western blot using a TMEFF2 antibody. **B)** TMEFF2 localization was analyzed in cells treated with DHT, or DMSO as a control, using confocal microscopy. Fixed 22Rv1 cells expressing endogenous TMEFF2 (green) were analyzed using a TMEFF2 antibody while RWPE1 cells transfected with a TMEFF2-myc-his tagged protein were analyzed using an anti-c-myc antibody. Preparations were mounted with medium containing DAPI (blue) for nuclear staining.
(TIF)

## References

[1] Heinlein CA, Chang C (2004) Androgen receptor in prostate cancer. Endocr Rev 25: 276–308. 15082523 10.1210/er.2002-0032

[2] Wang Q, Li W, Zhang Y, Yuan X, Xu K, et al (2009) Androgen receptor regulates a distinct transcription program in androgen-independent prostate cancer. Cell 138: 245–256. 19632176 10.1016/j.cell.2009.04.056 PMC2726827

[3] Liang T, Liao S (1975) A very rapid effect of androgen on initiation of protein synthesis in prostate. Proc Natl Acad Sci U S A 72: 706–709. 1054848 10.1073/pnas.72.2.706 PMC432384

[4] Liang T, Liao S (1975) Dihydrotestosterone and the initiation of protein synthesis by prostate ribosomes. J Steroid Biochem 6: 549–550. 1186241 10.1016/0022-4731(75)90033-3

[5] Heanue TA, Pachnis V (2006) Expression profiling the developing mammalian enteric nervous system identifies marker and candidate hirschsprung disease genes. Proc Natl Acad Sci U S A 103: 6919–6924. 16632597 10.1073/pnas.0602152103 PMC1458994

[6] Uchida T, Wada K, Akamatsu T, Yonezawa M, Noguchi H, et al (1999) A novel epidermal growth factor-like molecule containing two follistatin modules stimulates tyrosine phosphorylation of erbB-4 in MKN28 gastric cancer cells. Biochem Biophys Res Commun 266: 593–602. 10600548 10.1006/bbrc.1999.1873

[7] Afar DE, Bhaskar V, Ibsen E, Breinberg D, Henshall SM, et al (2004) Preclinical validation of anti-TMEFF2-auristatin E-conjugated antibodies in the treatment of prostate cancer. Mol Cancer Ther 3: 921–932. 15299075

[8] Gery S, Sawyers CL, Agus DB, Said JW, Koeffler HP (2002) TMEFF2 is an androgen-regulated gene exhibiting antiproliferative effects in prostate cancer cells. Oncogene 21: 4739–4746. 12101412 10.1038/sj.onc.1205142

[9] Glynne-Jones E, Harper ME, Seery LT, James R, Anglin I, et al (2001) TENB2, a proteoglycan identified in prostate cancer that is associated with disease progression and androgen independence. Int J Cancer 94: 178–184. 11668495 10.1002/ijc.1450

[10] Mohler JL, Morris TL, Ford OH,3rd, Alvey RF, Sakamoto C, et al (2002) Identification of differentially expressed genes associated with androgen-independent growth of prostate cancer. Prostate 51: 247–255. 11987153 10.1002/pros.10086

[11] Chen X, Overcash R, Green T, Hoffman D, Asch AS, et al (2011) The tumor suppressor activity of the transmembrane protein with epidermal growth factor and two follistatin motifs 2 (TMEFF2) correlates with its ability to modulate sarcosine levels. J Biol Chem 286: 16091–16100. 21393249 10.1074/jbc.M110.193805 PMC3091218

[12] Sreekumar A, Poisson LM, Rajendiran TM, Khan AP, Cao Q, et al (2009) Metabolomic profiles delineate potential role for sarcosine in prostate cancer progression. Nature 457: 910–914. 19212411 10.1038/nature07762 PMC2724746

[13] Morris DR, Geballe AP (2000) Upstream open reading frames as regulators of mRNA translation. Mol Cell Biol 20: 8635–8642. 11073965 10.1128/mcb.20.23.8635-8642.2000 PMC86464

[14] Sudhakar A, Ramachandran A, Ghosh S, Hasnain SE, Kaufman RJ, et al (2000) Phosphorylation of serine 51 in initiation factor 2 alpha (eIF2 alpha) promotes complex formation between eIF2 alpha(P) and eIF2B and causes inhibition in the guanine nucleotide exchange activity of eIF2B. Biochemistry 39: 12929–12938. 11041858 10.1021/bi0008682

[15] Vattem KM, Wek RC (2004) Reinitiation involving upstream ORFs regulates ATF4 mRNA translation in mammalian cells. Proc Natl Acad Sci U S A 101: 11269–11274. 15277680 10.1073/pnas.0400541101 PMC509193

[16] Palam LR, Baird TD, Wek RC (2011) Phosphorylation of eIF2 facilitates ribosomal bypass of an inhibitory upstream ORF to enhance CHOP translation. J Biol Chem 286: 10939–10949. 21285359 10.1074/jbc.M110.216093 PMC3064149

[17] Scheuner D, Song B, McEwen E, Liu C, Laybutt R, et al (2001) Translational control is required for the unfolded protein response and in vivo glucose homeostasis. Mol. Cell 7: 1165–1176. 10.1016/s1097-2765(01)00265-9 11430820

[18] Kim HJ, Park YI, Dong MS (2006) Comparison of prostate cancer cell lines for androgen receptor-mediated reporter gene assays. Toxicol in Vitro 20: 1159–1167. 16621434 10.1016/j.tiv.2006.03.003

[19] Esfahani A, Kendall CW, Bashyam B, Archer MC, Jenkins DJ (2010) The effect of physiological concentrations of sex hormones, insulin, and glucagon on growth of breast and prostate cells supplemented with unmodified human serum. In Vitro Cell Dev Biol Anim 46: 856–862. 20927603 10.1007/s11626-010-9351-x

[20] Baird TD, Wek RC (2012) Eukaryotic initiation factor 2 phosphorylation and translational control in metabolism. Adv Nutr 3: 307–321. 22585904 10.3945/an.112.002113 PMC3649462

[21] Aktas H, Fluckiger R, Acosta JA, Savage JM, Palakurthi SS, et al (1998) Depletion of intracellular Ca2+ stores, phosphorylation of eIF2alpha, and sustained inhibition of translation initiation mediate the anticancer effects of clotrimazole. Proc Natl Acad Sci U S A 95: 8280–8285. 9653178 10.1073/pnas.95.14.8280 PMC20967

[22] Masek T, Valasek L, Pospisek M (2011) Polysome analysis and RNA purification from sucrose gradients. Methods Mol Biol 703: 293–309. 21125498 10.1007/978-1-59745-248-9_20

[23] Harding HP, Novoa I, Zhang Y, Zeng H, Wek R, et al (2000) Regulated translation initiation controls stress-induced gene expression in mammalian cells. Mol Cell 6: 1099–1108. 11106749 10.1016/s1097-2765(00)00108-8

[24] Bennett NC, Gardiner RA, Hooper JD, Johnson DW, Gobe GC (2010) Molecular cell biology of androgen receptor signalling. Int J Biochem Cell Biol 42: 813–827. 19931639 10.1016/j.biocel.2009.11.013

[25] Pascall JC (1997) Post-transcriptional regulation of gene expression by androgens: Recent observations from the epidermal growth factor gene. J Mol Endocrinol 18: 177–180. 9195471 10.1677/jme.0.0180177

[26] Nantermet PV, Xu J, Yu Y, Hodor P, Holder D, et al (2004) Identification of genetic pathways activated by the androgen receptor during the induction of proliferation in the ventral prostate gland. J Biol Chem 279: 1310–1322. 14576152 10.1074/jbc.M310206200

[27] El-Awady MK, El-Garf W, El-Houssieny L (2004) Steroid 5alpha reductase mRNA type 1 is differentially regulated by androgens and glucocorticoids in the rat liver. Endocr J 51: 37–46. 15004407 10.1507/endocrj.51.37

[28] Fletcher CE, Dart DA, Sita-Lumsden A, Cheng H, Rennie PS, et al (2012) Androgen-regulated processing of the oncomir MiR-27a, which targets prohibitin in prostate cancer. Hum Mol Genet 21: 3112–3127. 22505583 10.1093/hmg/dds139

[29] Rajabi H, Joshi MD, Jin C, Ahmad R, Kufe D (2011) Androgen receptor regulates expression of the MUC1-C oncoprotein in human prostate cancer cells. Prostate 71: 1299–1308. 21308711 10.1002/pros.21344 PMC4916770

[30] Ramberg H, Eide T, Krobert KA, Levy FO, Dizeyi N, et al (2008) Hormonal regulation of beta2-adrenergic receptor level in prostate cancer. Prostate 68: 1133–1142. 18454446 10.1002/pros.20778

[31] Hamdi MM, Mutungi G (2011) Dihydrotestosterone stimulates amino acid uptake and the expression of LAT2 in mouse skeletal muscle fibres through an ERK1/2-dependent mechanism. J Physiol 589: 3623–3640. 21606113 10.1113/jphysiol.2011.207175 PMC3167122

[32] Xu Y, Chen SY, Ross KN, Balk SP (2006) Androgens induce prostate cancer cell proliferation through mammalian target of rapamycin activation and post-transcriptional increases in cyclin D proteins. Cancer Res 66: 7783–7792. 16885382 10.1158/0008-5472.CAN-05-4472

[33] Rutkowski DT, Arnold SM, Miller CN, Wu J, Li J, et al (2006) Adaptation to ER stress is mediated by differential stabilities of pro-survival and pro-apoptotic mRNAs and proteins. PLoS Biol 4: e374. 17090218 10.1371/journal.pbio.0040374 PMC1634883

[34] Pavitt GD, Ron D (2012) New insights into translational regulation in the endoplasmic reticulum unfolded protein response. Cold Spring Harb Perspect Biol 4: a01227810 DOI:10.1101/cshperspect.a012278. 10.1101/cshperspect.a012278 PMC3367556 22535228

[35] Clemens MJ (2001) Initiation factor eIF2 alpha phosphorylation in stress responses and apoptosis. Prog Mol Subcell Biol 27: 57–89. 11575161 10.1007/978-3-662-09889-9_3

[36] Haren MT, Siddiqui AM, Armbrecht HJ, Kevorkian RT, Kim MJ, et al (2011) Testosterone modulates gene expression pathways regulating nutrient accumulation, glucose metabolism and protein turnover in mouse skeletal muscle. Int J Androl 34: 55–68. 20403060 10.1111/j.1365-2605.2010.01061.x

[37] Yoshioka M, Boivin A, Ye P, Labrie F, St-Amand J (2006) Effects of dihydrotestosterone on skeletal muscle transcriptome in mice measured by serial analysis of gene expression. J Mol Endocrinol 36: 247–259. 16595697 10.1677/jme.1.01964

[38] Dai R, Yan D, Li J, Chen S, Liu Y, et al (2012) Activation of PKR/eIF2alpha signaling cascade is associated with dihydrotestosterone-induced cell cycle arrest and apoptosis in human liver cells. J Cell Biochem 113: 1800–1808. 22228470 10.1002/jcb.24051

[39] Liu S, Navarro G, Mauvais-Jarvis F (2010) Androgen excess produces systemic oxidative stress and predisposes to beta-cell failure in female mice. PLoS One 5: e11302. 20585581 10.1371/journal.pone.0011302 PMC2892018

[40] Ripple MO, Hagopian K, Oberley TD, Schatten H, Weindruch R (1999) Androgen-induced oxidative stress in human LNCaP prostate cancer cells is associated with multiple mitochondrial modifications. Antioxid Redox Signal 1: 71–81. 11225734 10.1089/ars.1999.1.1-71

[41] Sun XY, Donald SP, Phang JM (2001) Testosterone and prostate specific antigen stimulate generation of reactive oxygen species in prostate cancer cells. Carcinogenesis 22: 1775–1780. 11698338 10.1093/carcin/22.11.1775

[42] Mehraein-Ghomi F, Lee E, Church DR, Thompson TA, Basu HS, et al (2008) JunD mediates androgen-induced oxidative stress in androgen dependent LNCaP human prostate cancer cells. Prostate 68: 924–934. 18386285 10.1002/pros.20737

[43] Lozon TI, Eastman AJ, Matute-Bello G, Chen P, Hallstrand TS, et al (2011) PKR-dependent CHOP induction limits hyperoxia-induced lung injury. Am J Physiol Lung Cell Mol Physiol 300: L422–L429. 21186267 10.1152/ajplung.00166.2010 PMC3064291

[44] Foradori CD, Weiser MJ, Handa RJ (2008) Non-genomic actions of androgens. Front Neuroendocrinol 29: 169–181. 18093638 10.1016/j.yfrne.2007.10.005 PMC2386261

[45] Srivastava SP, Davies MV, Kaufman RJ (1995) Calcium depletion from the endoplasmic reticulum activates the double-stranded RNA-dependent protein kinase (PKR) to inhibit protein synthesis. J Biol Chem 270: 16619–16624. 7622470 10.1074/jbc.270.28.16619

[46] Pan C, Giraldo GS, Prentice H, Wu JY (2010) Taurine protection of PC12 cells against endoplasmic reticulum stress induced by oxidative stress. J Biomed Sci 17 Suppl 1S17 DOI:10.1186/1423-0127-17-S1-S17. 20804591 10.1186/1423-0127-17-S1-S17 PMC2994405

[47] Lin K, Taylor JR Jr, Wu TD, Gutierrez J, Elliott JM, et al (2011) TMEFF2 is a PDGF-AA binding protein with methylation-associated gene silencing in multiple cancer types including glioma. PLoS One 6: e18608. 21559523 10.1371/journal.pone.0018608 PMC3084709

[48] Juntti SA, Tollkuhn J, Wu MV, Fraser EJ, Soderborg T, et al (2010) The androgen receptor governs the execution, but not programming, of male sexual and territorial behaviors. Neuron 66: 260–272. 20435002 10.1016/j.neuron.2010.03.024 PMC2923659

[49] Lonergan PE, Tindall DJ (2011) Androgen receptor signaling in prostate cancer development and progression. J Carcinog 10: 20 DOI:10.4103/1477-3163.83937. 21886458 10.4103/1477-3163.83937 PMC3162670

[50] Chen TR, Wang P, Carroll LK, Zhang YJ, Han BX, Wang F (2012) Generation and characterization of Tmeff2 mutant mice. Biochem Biophys Res Commun 425: 189–194. 22828515 10.1016/j.bbrc.2012.07.064 PMC3428475

[51] Kumarasamy S, Gopalakrishnan K, Toland EJ, Yerga-Woolwine S, Farms P, et al (2011) Refined mapping of blood pressure quantitative trait loci using congenic strains developed from two genetically hypertensive rat models. Hypertension Res 34: 1263–1270. 10.1038/hr.2011.116 PMC3808166 21814219

[52] Lizotte E, Grandy SA, Tremblay A, Allen BG, Fiset C (2009) Expression, distribution and regulation of sex steroid hormone receptors in mouse heart. Cell Physiol Biochem. 23: 75–86. 10.1159/000204096 19255502

[53] Pausova Z, Abrahamowicz M, Mahboubi A, Syme C, Leonard GT, et al (2010) Functional variation in the androgen-receptor gene is associated with visceral adiposity and blood pressure in male adolescents. Hypertension 55: 706–714. 20083725 10.1161/HYPERTENSIONAHA.109.146720

[54] Ikeda Y, Aihara K, Yoshida S, Akaike M, Matsumoto T (2012) Effects of androgens on cardiovascular remodeling. J Endocrinol 214: 1–10. 22493003 10.1530/JOE-12-0126

[55] Floryk D, Kurosaka S, Tanimoto R, Yang G, Goltsov A, et al (2011) Castration-induced changes in mouse epididymal white adipose tissue. Mol Cell Endocrinol 345: 58–67. 21782885 10.1016/j.mce.2011.07.011 PMC3867123

[56] Quinkler M, Bujalska IJ, Kaur K, Onyimba CU, Buhner S, et al (2005) Androgen Receptor-Mediated Regulation of the α-Subunit of the Epithelial Sodium Channel in Human Kidney. Hypertension 46: 787–798. 16172422 10.1161/01.HYP.0000184362.61744.c1

[57] Cuesta R, Gupta M, Schneider RJ (2009) The regulation of protein synthesis in cancer. Prog Mol Biol Transl Sci 90: 255–292. 20374744 10.1016/S1877-1173(09)90007-2

[58] Silvera D, Formenti SC, Schneider RJ (2010) Translational control in cancer. Nat Rev Cancer 10: 254–266. 20332778 10.1038/nrc2824

[59] Mounir Z, Krishnamoorthy JL, Robertson GP, Scheuner D, Kaufman RJ, et al (2009) Tumor suppression by PTEN requires the activation of the PKR-eIF2alpha phosphorylation pathway. Sci Signal 2: ra85 DOI:10.1126/scisignal.2000389. 20029030 10.1126/scisignal.2000389 PMC3684442

[60] Gregory CW, He B, Johnson RT, Ford OH, Mohler JL, et al (2001) A mechanism for androgen receptor-mediated prostate cancer recurrence after androgen deprivation therapy. Cancer Res 61: 4315–4319. 11389051

[61] Putluri N, Shojaie A, Vasu VT, Nalluri S, Vareed SK, et al (2011) Metabolomic profiling reveals a role for androgen in activating amino acid metabolism and methylation in prostate cancer cells. PLoS One 6: e21417. 21789170 10.1371/journal.pone.0021417 PMC3138744

[62] Kinoshita H, Shi Y, Sandefur C, Meisner LF, Chang C, et al (2000) Methylation of the androgen receptor minimal promoter silences transcription in human prostate cancer. Cancer Res 60: 3623–3630. 10910077

[63] Gaughan L, Stockley J, Wang N, McCracken SRC, Treumann A, et al (2011) Regulation of the androgen receptor by SET9-mediated methylation. Nucleic Acids Res 39: 1266–1279. 20959290 10.1093/nar/gkq861 PMC3045589

[64] Ko S, Ahn J, Song CS, Kim S, Knapczyk-Stwora K, et al (2011) Lysine methylation and functional modulation of androgen receptor by Set9 methyltransferase. Mol Endocrinol 25: 433–444. 21273441 10.1210/me.2010-0482 PMC3045741

[65] Zhao JC, Yu J, Runkle C, Wu L, Hu M, et al (2012) Cooperation between Polycomb and androgen receptor during oncogenic transformation. Genome Res 22: 322–331. 22179855 10.1101/gr.131508.111 PMC3266039

