# Supplementary figures and images for: Androgen Signaling Promotes Translation of TMEFF2 in Prostate Cancer Cells via Phosphorylation of the α Subunit of the Translation Initiation Factor 2
Source: PLoS One. 2013 Feb 6;8(2):e55257. doi: 10.1371/journal.pone.0055257 (PMC3566213; doi:10.1371/journal.pone.0055257)

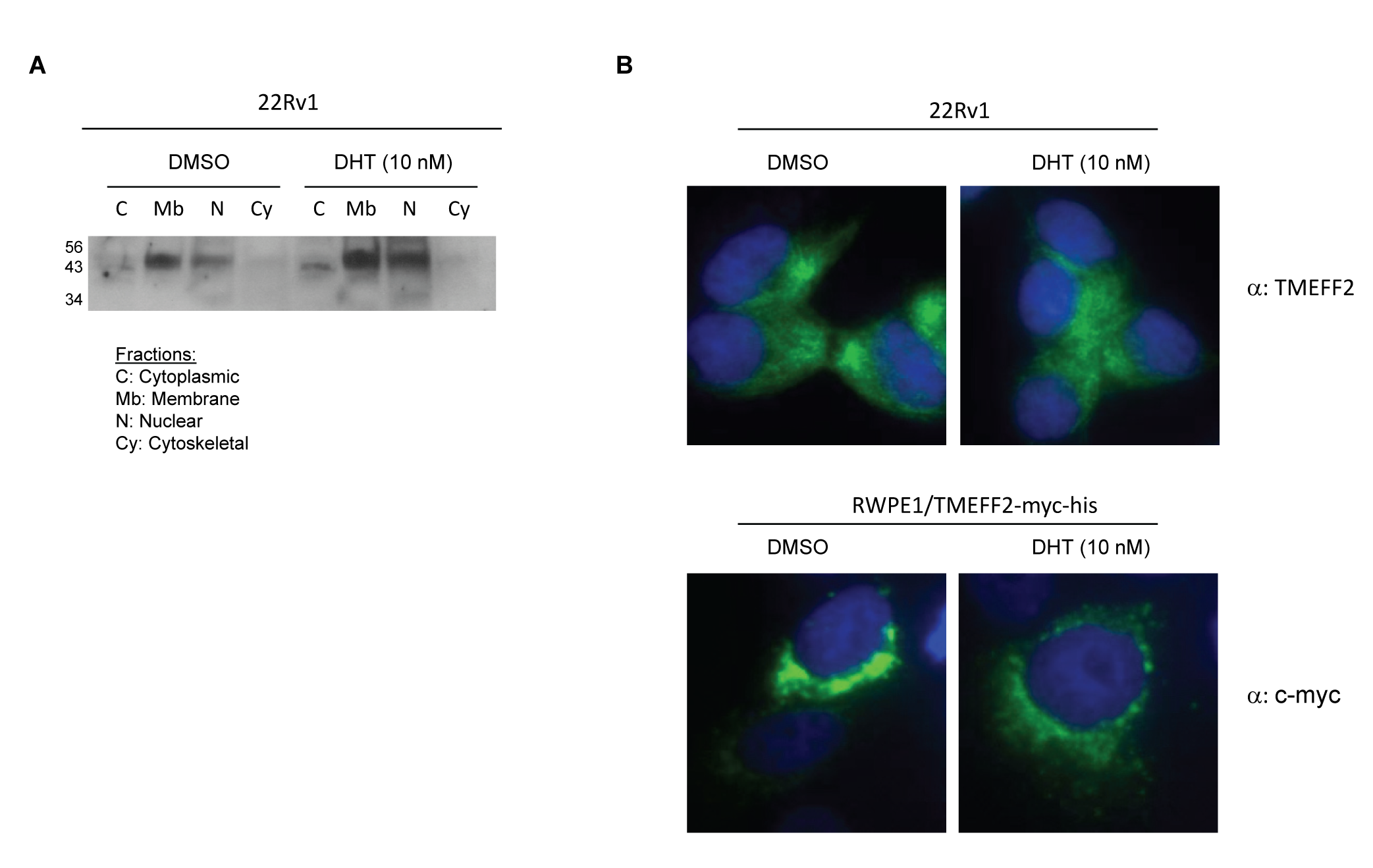

Supplement: Figure S1 — DHT does not alter the subcellular localization of TMEFF2. The potential effect of DHT on TMEFF2 localization was examined by subcellular fractionation (A) or immunofluorescence (B). A) 22Rv1 cells were treated with DHT or the vehicle control and subcellular fractions separated and analyzed by western blot using a TMEFF2 antibody. B) TMEFF2 localization was analyzed in cells treated with DHT, or DMSO as a control, using confocal microscopy. Fixed 22Rv1 cells expressing endogenous TMEFF2 (green) were analyzed using a TMEFF2 antibody while RWPE1 cells transfected with a TMEFF2-myc-his tagged protein were analyzed using an anti-c-myc antibody. Preparations were mounted with medium containing DAPI (blue) for nuclear staining. (TIF) [file pone.0055257.s001.tif]
